# A rapid review of the avian influenza PB2 E627K mutation in human infection studies

**DOI:** 10.14745/ccdr.v51i04a04

**Published:** 2025-04-03

**Authors:** Aaron MacCosham, Alexandra G Vasiliu, Nicole Atchessi

**Affiliations:** 1 Public Health Agency of Canada, Ottawa, ON

**Keywords:** avian influenza, E627K, human infection

## Abstract

**Background:**

The current avian influenza A(H5N1) epizootic poses a significant threat to public health, with sporadic infections in humans raising concerns about potential adaptation for efficient human transmission. Laboratory studies have provided evidence that the polymerase basic protein 2 (PB2) E627K mutation facilitates more efficient replication in mammals and humans. This mutation has been detected in Canadian poultry, wild birds and mammals.

**Objective:**

Our objective was to summarize the current state of evidence on the impact of the avian influenza PB2 E627K mutation on human adaptation, transmission, epidemiology and clinical outcomes in natural human infections.

**Methods:**

We employed a search strategy across MEDLINE, Embase, Scopus, Global Health and CAB Abstracts for articles published from each database’s inception until mid-May 2023.

**Results:**

We identified nine eligible articles for review that addressed human transmission or adaptation (n=5), epidemiological or clinical implication (n=1) or both topics (n=3). Some studies suggested that the PB2 E627K mutation may play a role in zoonotic transmission from birds to humans, with studies indicating its association with parallel evolution and positive selection in A(H5) and A(H7) viruses. Other studies presented analyses that supported the notion of an increased fatality rate among cases with the PB2 E627K mutation, highlighting its potential role as a virulence factor.

**Conclusion:**

The association of the PB2 E627K mutation with human adaptation, transmission and increased fatality rates highlights the importance of genomic surveillance under One Health umbrella. Further research is warranted to explore the role of this mutation and determine how it interacts with other mutations.

## Introduction

Avian influenza is a disease caused as a result of infection with influenza type A viruses. In birds, the virus primarily replicates in the respiratory and gastrointestinal tracts. The virus can cause sporadic infections in mammals, including when it crosses species barriers from birds ((1)), and, more recently, for the first time in mammals such as dairy cows ((2)). Such spillovers into humans can potentially have profound consequences. Even in the absence of sustained human-to-human transmission, avian influenza can pose significant threats and impact public health as A(H5) and A(H7) viruses have case fatality rates (CFR) of up to 52% and 39%, respectively ((3–5)). Although evidence of sustained human-to-human spread has not been reported, occasional avian influenza infections among humans have occurred, exemplified by the A(H5N1) and A(H7N9) subtypes ((6,7)). Humans can become infected with avian influenza through exposure to either live or dead birds infected with the virus, or through contaminated environments (e.g., poultry barns, live bird markets). Each zoonotic transmission and mammalian infection presents an opportunity for the virus to adapt as a result of random mutations or reassortment with other viruses, acquiring the ability to efficiently transmit between mammals including humans.

As of May 2024, 911 human cases of A(H5N1) have been reported worldwide since the emergence of the virus in humans in 1997 ((5)). The majority of these cases had known exposure to infected birds or their secretions ((8)). Since 2020, an epizootic of the avian influenza A(H5N1) virus has been occurring in wild and domestic birds in Europe, Africa and Asia. The virus was later detected in Newfoundland, Canada, in late 2021 ((9)) and detections and outbreaks have been observed in Canadian provinces and territories, as well as across the United States, Mexico and throughout Central and South America since then ((10,11)). As of May 2024, no domestically acquired human A(H5N1) infections have been reported in Canada; however, a glutamic acid to lysine mutation at the 627 residue (E627K) in polymerase basic protein 2 (PB2) has been detected in Canadian wildlife ((12)). This mutation was detected in domestic poultry in Canada for the first time in April 2023 ((13)). Laboratory studies of the E627K mutation provide evidence that it can lead to avian influenzas more efficiently replicating in mammals, as well as mammalian, including human, cells ((14–16)). Currently, there is a lack of literature reviews on the impact of the avian influenza PB2 E627K mutation in humans. Considering that A(H5N1) and other avian influenza viruses have already spilled over on numerous occasions, it is imperative to gain a better understanding of the biological and clinical implications in humans infected with an avian influenza virus bearing the PB2 E627K mutation. In addition, a rapid review of this topic would aid ongoing risk assessments by clarifying current knowledge and identifying relevant aspects from a human perspective. The two research questions we sought to address were as follows: 1) What is the impact of the avian influenza virus PB2 E627K mutation on human adaptation and transmission?; and 2) What is the current state of evidence, as of May 16, 2023, on the epidemiology and clinical implications of the avian influenza virus PB2 E627K mutation in human infections?

## Methods

### Search strategy and selection criteria

In collaboration with a Health Canada librarian, we developed a search strategy and searched the MEDLINE (1946 to May 17, 2023), Embase (1974 to May 16, 2023), Scopus (1970 to May 18, 2023), Global Health (1973 to Week 19 2023) and CAB Abstracts (1973 to Week 19 2023) databases for published studies. We restricted our search to articles published in either English or French. No publication year restrictions were applied to capture the maximum number of articles, as we expected a low number of articles on this topic. The following combination of keywords and MeSH terms were used: “E627K,” “human,” “exp persons,” “exp people,” “exp human experimentation,” “exp human development,” “human embryo,” “exp ‘named groups of persons’,” “exp human experimentation” (**Appendix**, **Table A1**). Furthermore, we searched grey literature for additional articles.

We included studies providing empirical evidence on the impact of the E627K mutation in the PB2 protein in human infections of any avian influenza. Studies were excluded if the unit of observation was not the case or case’s sample, such as a study using cells as the unit of observation. We used DistillerSR, an online application designed for the screening phases of a literature review, for primary and secondary screening and data extraction. The primary screen of titles and abstracts was independently done in duplicate. All articles with conflicting inclusion/exclusion decisions were included for secondary screening to ensure all relevant studies received thorough consideration. The secondary screen of full-text articles was also independently performed in duplicate. We resolved any articles in disagreement during secondary screening and reached a consensus on the final selection of articles. We omitted a critical appraisal of study quality in this rapid review due to time constraints.

## Results

**Figure 1:**
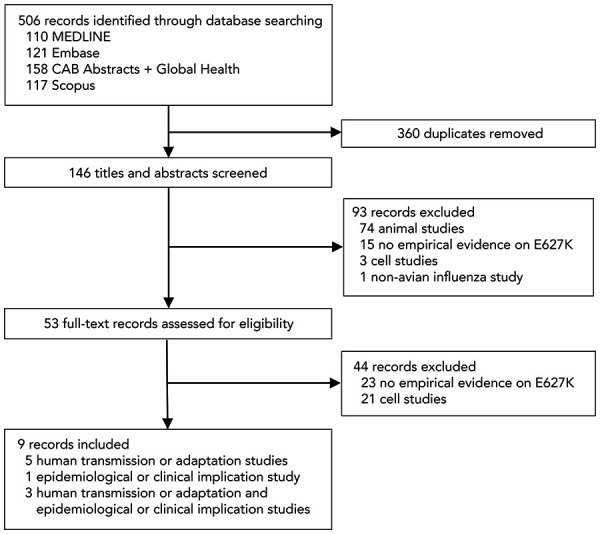
Flow chart of search results and study selection process

**Figure 1** shows the summary of the study selection process. Our search strategy yielded 506 articles. After removing 360 duplicates, we screened the title and abstract of 146 articles and excluded 93 articles, with 74 excluded because they were animal studies, 15 due to a lack of empirical evidence on the PB2 E627K mutation, three because they were cell studies and one because it was a non-avian influenza study. We performed a full-text review of 53 articles and excluded 44 articles, 23 of which lacked empirical evidence on the PB2 E627K mutation and 21 of which were cell studies. This resulted in a final selection of nine articles that addressed topics related to human adaptation or transmission (n=5) ((17–21)), epidemiological or clinical implication (n=1) ((22)) or both subjects (n=3) ((23–25)) (**Table 1**).

**Table 1:** Characteristics and summary of findings from articles included in the study

First author, year	Objectives of the study	Pathogen targeted	Sample size (human host)	Increased adaptation and/or transmission	Epidemiological and/or clinical implication
Guo *et al.*, 2019	Identify genetic changes that occurred during avian influenza H5N1 viruses’ multiple invasions to humans	A(H5N1)	Not specified	The authors interpreted their finding as the E627K mutation potentially aiding A(H5Nx) viruses to spillover into humans	Not reported
Moncla *et al.*, 2020	Examine how H5N1 viruses evolve during spillover in humans and ducks	A(H5N1)	N=7	Yes The authors suggested that PB2 E627K and HA A150V were mutations in their study that were likely human adaptive	Not reported
Jonges *et al.*, 2014	Investigate whether PB2 E627K and K416R mutations occurred in the avian or a human host during a large H7N7 outbreak in the Netherlands	A(H7N7)	N=1	Yes The author provided evidence of the PB2 E627K mutation’s role in parallel evolution and positive selection^a^	Not reported
Liu *et al.*, 2020	Provide beneficial data for understanding the influenza-host interaction and the adaptation of H7N9 in humans	A(H7N9)	N=38	Yes The authors suggested the occurrence of dynamic viral adaptation during infection in humans	Yes
Sha *et al.*, 2016	Compare the molecular epidemiology and virology of avian influenza A (H7N9) viruses from mild, severe and fatal cases	A(H7N9)	N=99	Not reported	Yes
Wang *et al.*, 2014	Compared the signature amino acids of avian influenza A(H7N9) viruses from human and non-human hosts and analyzed the reassortants of 146 influenza A(H7N9) viruses with full genome sequences	A(H7N9)	N=103	Yes The author provided evidence that the E627K mutation was being positively selected in humans	Yes
Gao *et al.*, 2014	Report information on the clinical features of two patients from the same family infected with H7N9 virus, the genomic sequences of the viruses harboured and antiviral drug sensitivity	A(H7N9)	N=2	Yes A(H7N9) human-to-human transmission may have occurred via unprotected contact. PB2 E627K mutation may have had a role in the probable human-to-human transmission. However there is a possibility that other PB2 mutations may have played a role (K191E, V511I, M535L, M570I, I647V and K702R)	Yes
Xiang *et al.*, 2018	Determine whether adaptive evolution had occurred in human-isolated H7N9 viruses	A(H7N9)	Not specified	Yes E627K mutation underwent parallel evolution, which might play a role in crossing the species barrier from avian hosts into humans	Not reported
Zou *et al.*, 2018	Describe dynamic variation of signature amino acids of H7N9 influenza A virus during disease development	A(H7N9)	N=11	Yes The authors found that PB2 627 and NA 292 were molecular “hot spots” displaying vigorous variation, with the E627K substitution exhibiting dynamic variation during human infection	Not reported

Abbreviations: HA, hemagglutinin protein; NA, neuraminidase; PB2, polymerase basic protein 2

^a^ Parallel evolution is when similar mutations independently appear indicating adaptive changes while positive selection is the increase in the frequency of advantageous mutations pointing to important amino acid changes for adapting to a new host

### PB2 E627K mutation: Human adaptation and transmission

Human adaptation of an avian influenza virus can be defined as its acclimation to operate optimally in a human ((26)). Seven studies ((17–23)) reported human adaptation of the avian influenza virus carrying the PB2 E627K mutation in infected persons, in addition to five studies on A(H7) viruses and two studies on A(H5) viruses. Within the A(H7) studies, Jonges *et al*. ((17)) observed the emergence of the PB2 E627K mutation during a single human A(H7N7) infection. The PB2 E627K mutation was not detected in the two source farm samples or the control farm sample, but it was detected in all three human samples taken at different time points. These findings underscore the significance of minimizing human exposure to avian influenza viruses in order to reduce the probability of the virus adapting to humans. Two of the A(H7) studies provided evidence of the PB2 E627K mutation’s role in parallel evolution and positive selection. Parallel evolution is when similar mutations independently appear, indicating adaptive changes, while positive selection is the increase in the frequency of advantageous mutations, pointing to important amino acid changes for adapting to a new host ((18)). Xiang *et al*. ((18)) identified 34 mutation sites in human-isolated A(H7N9) viruses as being significant signals for parallel evolution. They identified nine PB2 parallel amino acid sites. The PB2 mutations sites, T106A, Q591K, E627K and D701N, were shared on three or more phylogenetic branches from avian to human hosts, with the E627K mutation being shared on the most branches. The authors interpreted this as meaning the E627K mutation may have undergone parallel evolution to potentially play a role in crossing the species barrier from avian hosts into humans. Similarly, Wang *et al*. ((24)) found that there were significantly more non-synonymous than synonymous nucleotide substitutions at the PB2 627 location. In the context of the PB2 627 site, a synonymous substitution would be one that leads to a glutamic acid (E) and a non-synonymous substitution would be one that leads to a lysine (K). They argued that this provided evidence that the mutation was being positively selected for in humans.

Two (H7) studies examined the PB2 E627K mutation’s role during human infection. Zou *et al*. ((19)) observed dynamic evolution of the A(H7N9) virus during the infection of some human cases. Dynamic in this context refers to the change of one amino acid residue to another at a given location, which is a sign of adaptation. PB2 627 and NA 292 were molecular “hot spots” displaying vigorous variation, with the PB2 E627K substitution exhibiting dynamic variation. Liu *et al*. ((25)) further delved into the dynamic interspecies adaptation in A(H7N9) viruses. They suggested that dynamic viral adaptation during infection, also called “genetic tuning,” had occurred in this case. However, the authors reported no correlation between the ratio of K at the PB2 627 location and the number of days after disease onset, indicating that the viral samples in the study did not demonstrate adaptation over time through the ratio of K at this location. They put forth the idea that this may reflect diverse adaptation processes.

The A(H7) studies’ findings were similar to those from the A(H5) studies. Guo *et al*. ((25)) identified 126 parallel evolution mutations on A(H5Nx) host-shift branches. They observed the PB2 E627K mutation on 30/171 (17.5%) phylogenetic branches from avian to human hosts, which was the highest number among all the mutations the authors identified and 20 more than the second most observed mutation, PB2 D701N. The authors interpreted this finding as the E627K mutation potentially aiding A(H5Nx) viruses to spillover into humans. Like Guo *et al*., Moncla *et al*. ((21)) reported that the transition of E to K in the PB2 protein at the 627 site was more commonly found on phylogenetic branches that led to human infections. The authors suggested that PB2 E627K and HA A150V were mutations that were likely human adaptive.

We identified one study ((23)) that reported potential human transmission of avian influenza. The authors investigated two A(H7N9) cases within a familial cluster in Zhejiang, China, in 2013, both of which were infected with viruses exhibiting the PB2 E627K mutation. They suggested that A(H7N9) human-to-human transmission may have occurred via unprotected contact. Their reasoning stems from three factors: the confirmed A(H7N9) infections in both cases, the strong epidemiological link between them, and the striking similarities in the nature of the viruses extracted, including their mutations. Although the authors suggest that the PB2 E627K mutation may have had a role in the probable human-to-human transmission, several other PB2 mutations (i.e., K191E, V511I, M535L, M570I, I647V and K702R), which were not present in the first A(H7N9) epidemic wave in China, could have played a role.

### E627K mutation: Epidemiological and clinical description

Four articles touched on the epidemiological or clinical implications of the avian influenza PB2 E627K mutation in humans. The previously described Gao *et al.* study ((23)) also reported on the epidemiological and clinical implications on the two A(H7N9) cases with the PB2 E627K mutation. In this study, the index case, a 57-year-old male with a history of hypertension, resided in a semi-urban area of Anji County, Zhejiang Province, China. Notably, he purchased food every day from a nearby wet market where various live poultry were sold. He visited a rural area and cleaned a chicken coop on November 16, 2013. He developed symptoms five days later and the laboratory results confirmed his A(H7N9) infection on November 25, which is also when he started to receive oseltamivir. His symptoms included fever, shortness of breath and productive cough. He was later diagnosed with pneumonia and acute respiratory distress syndrome, and subsequently died on an unspecified date.

The second case, a 31-year-old male businessman and the index case’s son-in-law, had close contact with the index case, including providing care to him without personal protective equipment, between November 20 and November 26. He started experiencing symptoms on November 30 and the laboratory results confirmed his A(H7N9) infection on December 5. He reported no exposure to poultry or any animal in the two weeks prior to illness onset. His symptoms included fever, shortness of breath, a productive cough and diarrhea. An outpatient clinic provided the case with oseltamivir therapy once and, upon admission, the medical staff provided the case with peramivir on the day his A(H7N9) infection was confirmed. He was diagnosed with pneumonia but was later discharged from the hospital and made a full recovery. To our knowledge, this may be one of the rare, documented cases of probable human-to-human transmission involving the PB2 E627K.

Sha *et al*. ((22)) examined genetic mutations of A(H7N9) viruses from human cases and reported their clinical information in their study. Among the 83 cases for which sequencing results at the PB2 627 site and case severity information were available, the E627K mutation was detected in three out of nine cases (33.3%) with mild symptoms, 30 out of 44 cases (68.2%) with severe symptoms and 26 out of 30 cases (86.7%) that resulted in fatalities. The PB2 E627K mutation was associated with a slight increase in CFR in the study. The authors indicated that advanced age, delays in confirming diagnosis and delays in starting antiviral treatment were the primary contributing factors to an increased risk of mortality. Additionally, mutations NA R294K and PA V100A were individually linked to a higher fatality rate.

In addition to providing evidence for human adaptation, Wang *et al*. ((24)) also assessed the association between A(H7N9) viral mutations in human cases and clinical outcome. The CFR among those infected with A(H7N9) viruses with a K at the PB2 627 site was 53% (n=19/36), while it was 29% (n=4/14) for those with an E at the same location. The authors reported that although most mutations did not impact the CFR, only the PB2 E627K mutation slightly increased the CFR, but without statistical significance using a corrected chi-square analysis.

Liu *et al*. ((25)) examined the adaptation patterns of the PB2 E627K mutation in A(H7N9) viruses and its relationship with disease severity. The authors compared early adaptation of E627K (assessed via lysine K ratios per days post onset of disease) in the upper respiratory tract of deceased cases versus recovered cases. They determined that fatal cases had a more rapid adaptation of the E627K mutation compared to those that recovered. In this case, rapid adaptation can be described as having higher lysine K ratios per days post onset of disease during the acute phase of the infection when samples were collected. The authors posited that their findings indicated an association between the genetic tuning of the PB2 E627K mutation and the pathogenicity of A(H7N9) during human infection.

All these findings suggest that the PB2 E627K mutation may be linked to increased virulence of the virus.

## Discussion

Our rapid review of articles on human adaptation, transmission, epidemiology and clinical implications of the avian influenza virus PB2 E627K mutation compiles research focusing on its impact in human infections. We identified nine relevant studies ((17–25)), with eight describing the mutation and human transmission or adaptation and four describing the mutation and human epidemiology or clinical implications. The identified studies provided some evidence that the PB2 E627K mutation may play a role in the interspecies transmission of avian influenza from birds to humans. Studies examining human adaptation of A(H5) and A(H7) viruses provided evidence of parallel evolution ((18,25)) and positive selection ((24)) of the PB2 E627K mutation, suggesting it may be an adaptive change for crossing the species barrier.

The analysis of case severity across two studies ((22,24)) linked the PB2 E627K mutation to increased fatality rates, suggesting its potential role as a virulence factor. Advanced age, delays in diagnosis confirmation and delays in starting antiviral treatment were also identified as contributing factors to increased CFR, emphasizing the importance of timely interventions. One study ((25)) provided evidence that there are different adaptation patterns of the PB2 E627K mutation and that a more rapid adaptation of the PB2 E627K mutation was associated with fatal cases.

The PB2 E627K mutation was observed in two cases where human-to-human transmission was suspected, emphasizing the potential risk associated with this genetic change ((23)). For sustained transmission of avian influenza viruses in the human population, several key factors must occur ((27)). Firstly, effective exposure to the.avian influenza virus is essential. Secondly, the virus must develop the affinity to bind to the sialic acid receptors to infect human cells. Thirdly, the virus must acquire the ability to replicate within human cells. Lastly, the ability of the virus to exit the cells and human and be transmitted to another human host is critical. Therefore, a single PB2 E627K mutation might not be sufficient to lead to a sustained human-to-human transmission of avian influenza viruses A(H5) and A(H7).

## Limitations

We identified several limitations with our study. The first is that there are few publications on this topic in the current body of literature; more publications on the impact of the avian influenza PB2 E627K mutation in human infections would provide better evidence of the mutation’s impact. The scarcity of publications on this topic raises concerns about the possibility of publication bias, potentially resulting from the withholding of null results ((28)). Additionally, variations in specimen collection methods (pharyngeal swab vs. sputum vs. nasal lavage fluids) and the time of sample collection within the infection/symptomatic period (e.g., Day 1 vs. Day 5) may have influenced the results. Future studies should consider these factors to provide a more nuanced understanding of the dynamics of the PB2 E627K mutation. Another limitation is that the review captures studies published until May 16, 2023. Relevant studies may have been published since. The final limitation is that we did not perform an appraisal of quality of included studies.

## Conclusion

To our knowledge, this is the first study to review the published literature for the impact of the avian influenza PB2 E627K mutation in humans. Findings suggest that the PB2 E627K mutation may be linked to increased virulence of the virus. Additionally, this mutation could have played a role in the virus transitioning from avian hosts to humans. However, a single PB2 E627K mutation may not be enough to enable sustained human-to-human transmission of avian influenza viruses A(H5) and A(H7). Our review provides public health authorities with the available evidence as of May 16, 2023, on the potential implications of this mutation for risk assessments, should it emerge in mammals or humans in their jurisdiction. The PB2 E627K mutation’s potential role in human adaptation, transmission and clinical implications underscores the significance of genomic surveillance in humans and animals and timely information sharing from a One Health perspective. Additional studies are needed on the avian influenza PB2 E627K mutation, including those that compare the clinical and epidemiological characteristics of cases with the mutation and those without. Further studies should also review other mutations, such as the PB2 D701K mutation, both independently and in conjunction with the PB2 E627K mutation, to better understand the dynamics of avian influenza mutations in human infection.

AM — Conceptualization, data extraction, writing–original draft, writing–review & editing

AGV — Conceptualization, data extraction, writing–review & editing

NA — Conceptualization, writing–review & editing, supervision

The authors acknowledge the Health Canada Library for their assistance with a literature search on the research topic. The authors extend their gratitude to Simran Sandhu for her support in the literature screening process. The authors express their appreciation to Erin Leonard and Yohannes Berhane for their review of the manuscript.

## Appendix

**Table A1:** Search strategy to identify all included studies providing empirical evidence on the impact of the E627K mutation in the PB2 protein in human infections of any avian influenza

Database	MEDLINE	Embase	Scopus	Global Health and CAB Abstracts
Search date	May 17, 2023	May 16, 2023	May 18, 2023	Week 19 2023
Search terms	1: E627K.kw,kf,tw. 2: humans/ or exp persons/ or exp human experimentation/ 3: (human or humans or people or person* or patient? or inpatient* or volunteer* or participant* or man or men or woman or women or child* or p?ediatric* or neonate? or newborn* or new born* or baby or babies or infant* or toddler* or youth* or teen* or adolescen* or preteen* or preadolescen* or girl* or boy* or school age* or schoolage* or student* or adult* or senior* or elderly or homeless* or worker* or employee* or occupat* or first responder* or police* or paramedic* or firefighter* or consumer? or smoker* or aboriginal? or ((urban or rural or vulnerab* or low income or indigenous or high risk) adj2 (population? or group?)) or public or citizen? or Inuit* or First Nation* or Metis).tw,kf. 4: 2 or 3 [human] 5: 1 and 4	1: E627K.kw,kf,tw. 2: exp Human/ or exp human development/ or human embryo/ or exp "named groups of persons"/ 3: (human or humans or people or person* or patient? or inpatient* or volunteer* or participant* or man or men or woman or women or child* or p?ediatric* or neonate? or newborn* or new born* or baby or babies or infant* or toddler* or youth* or teen* or adolescen* or preteen* or preadolescen* or girl* or boy* or school age* or schoolage* or student* or adult* or senior* or elderly or homeless* or worker* or employee* or occupat* or first responder* or police* or paramedic* or firefighter* or consumer? or smoker* or aboriginal? or ((urban or rural or vulnerab* or low income or indigenous or high risk) adj2 (population? or group?)) or public or citizen? or Inuit* or First Nation* or Metis).tw,kf. 4: 2 or 3 [human] 5: 1 and 4	TITLE-ABS-KEY (e627k) AND TITLE-ABS-KEY (human OR humans OR people OR person* OR patient* OR inpatient* OR volunteer* OR participant* OR man OR men OR woman OR women OR child* OR pediatric* OR paediatric* OR neonate* OR newborn* OR “new born*” OR baby OR babies OR infant* OR toddler* OR youth* OR teen* OR adolescen* OR preteen* OR preadolescen* OR girl* OR boy* OR “school age*” OR schoolage* OR student* OR adult* OR senior* OR elderly OR homeless* OR worker* OR employee* OR occupat* OR “first responder*” OR police* OR paramedic* OR firefighter* OR consumer* OR smoker* OR aboriginal* OR ((urban OR rural OR vulnerab* OR “low income” OR indigenous OR “high risk”) W/1 (population* OR group*)) OR public OR citizen* OR inuit* OR “First Nation*” OR metis)	1: E627K.tw,id. 2: man/ or exp people/ or exp human experimentation/ 3: (human or humans or people or person* or patient? or inpatient* or volunteer* or participant* or man or men or woman or women or child* or p?ediatric* or neonate? or newborn* or new born* or baby or babies or infant* or toddler* or youth* or teen* or adolescen* or preteen* or preadolescen* or girl* or boy* or school age* or schoolage* or student* or adult* or senior* or elderly or homeless* or worker* or employee* or occupat* or first responder* or police* or paramedic* or firefighter* or consumer? or smoker* or aboriginal? or ((urban or rural or vulnerab* or low income or indigenous or high risk) adj2 (population? or group?)) or public or citizen? or Inuit* or First Nation* or Metis).tw,id. 4: 2 or 3 [human] 5: 1 and 4
